# Pyroptosis-related genes prognostic model for predicting targeted therapy and immunotherapy response in soft tissue sarcoma

**DOI:** 10.3389/fphar.2023.1188473

**Published:** 2023-05-05

**Authors:** Mengmeng Liu, Quan Li, Yao Liang

**Affiliations:** ^1^ State Key Laboratory of Oncology in South China, Collaborative Innovation Center for Cancer Medicine, Sun Yat-sen University Cancer Center, Guangzhou, China; ^2^ Department of Burn and Plastic Surgery, The Sixth Affiliated Hospital, South China University of Technology, Foshan, China; ^3^ Department of Gastric Surgery, Sun Yat-sen University Cancer Center, Guangzhou, China

**Keywords:** soft tissue sarcoma, pyroptosis, chemotherapy, immunotherapy, prognosis

## Abstract

Several studies have highlighted the potential of pyroptosis as a target for cancer treatment. This article focuses on the specific roles and clinical implications of pyroptosis-related genes (PRGs) in soft tissue sarcoma (STS). By analyzing differentially expressed PRGs in STS compared to normal tissue, our study evaluates the interactions, biological functions, and prognostic values of PRGs in STS. Through LASSO COX regression analysis, a five-gene survival related-risk score (PLCG1, PYCARD, CASP8, NOD1, and NOD2) was created, which examined both in TCGA cohort and training cohort (GSE21050, GSE30929, and GSE63157). Furthermore, we developed a nomogram incorporating clinic factors and the risk scores of the PRGs, which showed decent accuracy of prediction as evidenced by calibration curves. Additionally, our study analyzed the Tumor Immune Dysfunction and Exclusion Algorithm (TIDE) and IMvigor 210 cohorts to investigate the immunotherapy response, and found that immunotherapy was more beneficial for patients with minimal risk of PRGs than those exhibiting greater risk. Finally, GDSC and CAMP databases were used to screen for effective chemotherapy or targeted drugs that are sensitive to the high-risk populations, including doxorubicin, imatinib, and sorafenib. In conclusion, this study provides a comprehensive analysis of the PRG landscape in STS and constructs a novel risk model to predict prognosis and different therapeutic responses of STS patients, which is helpful for achieving precision medicine.

## 1 Introduction

As a form of solid malignant tumor arising from mesenchymal tissue, soft tissue sarcoma (STS) accounts for approximately 15% of malignancies amongst children and 1% amongst adults (Raney, 2002). The specific mechanism of its formation and progression remains unclear, likely due to the large number of subtypes and strong heterogeneity of STS. Furthermore, traditional treatment methods have limited therapeutic effects on advanced STS (Schuetze and Ray, 2005; Ray-Coquard et al., 2018). While emerging targeted therapies and immunotherapies, such as anlotinib, pazopanib, and immune checkpoint inhibitors (ICIs), have improved outcomes for STS patients in recent years (Chi et al., 2018; Gamboa et al., 2020; Zhu et al., 2020; Schmoll et al., 2021), the rate of efficacy remains modest, and only a few patients experience long-lasting effects. Therefore, precise biomarkers are urgently needed to categorize STS patients into various risk categories and select an appropriate treatment population for immunotherapy.

Pyroptosis is a unique type of programmed cell death that is initiated by caspase activation and leads to lysis and granzyme protease emission (Bergsbaken et al., 2009). Unlike traditional apoptosis, pyroptosis mainly relies on the activation of caspase-1/11 (Miao et al., 2010; Miao et al., 2011; Broz et al., 2012). Pyroptosis is mainly involved in inflammatory diseases and has been shown to be an important factor in the development of cardiovascular and cerebrovascular diseases, such as coronary atherosclerosis (Wang et al., 2020a). The intricate biological activities of pyroptosis in cancer have been revealed through a thorough understanding of cell pyroptosis (Wang et al., 2020b). For instance, nucleotide-binding domain-like receptor 3 (NLRP3) can enhance the malignant proliferation of lung cancer and lymphoma (Liang et al., 2020; Lu et al., 2021). A recent study found that PD-L1 can promote tumor progression by upregulating the expression of GSDMC, which is involved in pyroptosis, suggesting that pyroptosis may be related to tumor immune escape and providing new ideas for cancer prevention and treatment (Hou et al., 2020). However, it remains unclear whether genes related to pyroptosis affect the progression of STS.

In this study, the transcriptomes of STS patients were analyzed to identify pyroptosis-associated genetic markers. Based on the characteristics of pyroptosis-related genes (PRGs), two distinct subtypes of STS were identified. A comprehensive analysis was then conducted to investigate the potential association between various PRGs risk scores and clinical pathological data and immunological status. Subsequently, a nomogram incorporating clinical factors and PRG scores was developed to predict prognosis for STS patients. Additionally, the likelihood of benefit from anti-PD-L1 treatment for patients with specific PRG features was predicted. Finally, several potentially sensitive small molecule inhibitors for STS patients with different PRGs were proposed.

## 2 Materials and methods

### 2.1 Datasets

We retrieved the RNA sequencing (RNA-seq) profile of TCGA-SARC through the GDC API (https://portal.gdc.cancer.gov/repository). From this cohort, 263 STS and two typical soft tissue samples were obtained. From the GTEx database, 864 normal tissues (386 subcutaneous fatty tissue and 478 skeletal muscle) were obtained (https://xenabrowser.net/da-tapages) to assess the different PRGs between normal tissue and STS. For independent validation cohorts, including GSE21050, GSE30929, and GSE63157, whose RNA-seq and clinical data were retrieved from the Gene Expression Omnibus (GEO) database (https://www.ncbi.nlm.nih.gov/geo). The GSE21050 cohort was used to validate disease-free survival (DFS) time, whereas the GSE30929 and GSE63157 cohorts were utilized to assess overall survival (OS) time.

### 2.2 Identification of differentially expressed PRGs

Supplementary Table S1 displayed a list of PRGs. The expression data from the TCGA and GETx datasets were transformed into FPKM values before analyzing the differences. The “limma” R package detected PRGs between typical and tumor tissues. The differentially expressed PRGs were notated as follows: * if *p* < 0.05, ** if *p* < 0.01, and *** if *p* < 0.001 were used. The mutation landscape and the correlation between mRNA expression level with methylation rate were respectively analyzed by the “maftool” package and GSCA Lite.

### 2.3 Development and validation of the PRGs prognostic model

Moreover, the predictive significance of PRGs was examined using LASSO cox regression analysis for evaluating correlations among the genes as well as their status of survival in the TCGA-SARC cohort. Five genes associated with survival were chosen for further investigation. After centralization and standardization by using R packaeg “scale”, the risk score was determined. The formula for the risk score was as follows: $\sum i=Coefficient\left(mRNAi\right)\times Expression\left(mRNAi\right)$ . Additionally, Kaplan-Meier analysis was used to compare the OS times of the two groupings. Principal component analysis (PCA) was done using the “prcomp” function from the “stats” R package. The R packages “survival”, “survminer”, and “timeROC” were used to analyze 1-, 2-, 3-, and 5-year ROC curves.

Three SARC cohorts from the GEO database were used to validate the OS and DFS features (GSE21050, GSE30929, and GSE63157). To verify the particular model used to the TCGA-SARC cohort, the “scale” function was utilized in expression normalization for every PRG. Afterward, risk scores were calculated based on the usual technique in the TCGA cohort.

### 2.4 Independent prognostic analysis of the PRGs-risk score

The regression model was constructed using factors such as age, gender, tumor size (length, breadth, and depth), and PRGs-risk score. The investigation was carried out using univariate and multivariate cox regression models, with the findings shown using a nomogram. The R package “timeROC” was used to determine the predictive accuracy of the model, and then decision curve analysis (DCA) was used to determine net benefit (Vickers et al., 2008).

### 2.5 Evaluating the efficacy of immunotherapy in different groups

We used tumor immune dysfunction and exclusion (TIDE) and submap algorithms (https://cloud.genepattern.org/gp) to predict anti-PD-1 or anti–CTLA-4 response rates in STS patients with high PRGs-risk or low PRGs–risk scores. As an externally verified model for predicting immunotherapy response, The IMvigor210 transcriptome and clinical data were retrieved (http://research pub.gene.com/IMvigor210CoreBiologies) to confirm that the PRGs-risk model could predict anti-PD-L1 efficacy.

### 2.6 Predicting response to chemotherapy and targeted therapy

In addition, we used the biggest publicly available pharmacogenomics database, the Genomics of Drug Sensitivity in Cancer (GDSC, https://www.cancerrxgene.org/) (Yang et al., 2013), to analyze and forecast the response of the two groups of patients to the NCCN-approved medications for STS treatment. The pRRophetic package of the R software was used to predict the IC50 values of several medications for the two groups. A ten-fold cross-validation of the GDSC training model evaluated the accuracy of the drug sensitivity which predictive based on the PRGs-risk score.

### 2.7 Identification of potential small molecule drugs

As a database utilizing matching algorithms, connection Map (CMAP) examines the link between drugs, expression of genes, and changes in phenotypes (http://www.broadinstitute.org). We submitted the PRGs to the CMAP database in order to find possible small molecule inhibitors that could improve the prognosis of STS patents (Fabbri et al., 2021). Enrichment scores for small molecule medications were calculated on a scale of −1 to 1 to indicate the degree of similarity between the expression spectrums. A negative enrichment score and *p* < 0.05 indicated that the medication would be effective in the treatment of STS.

### 2.8 Cell cultures and quantitative real-time PCR

DMEM (Gibco, United States) supplemented with 10% fetal bovine serum (FBS) (Gibco, United States) cultured the following: human undifferentiated pleomorphic sarcoma cell line (U2197), human malignant embryonic rhabdomyosarcoma cell line (RD), and human lung fibroblasts (HLF). Specifically, those were cultured in a humidified incubator containing 5% carbon dioxide at 37 °C. TRIZOL reagent was used to extract all RNA from the cell lines (SigmaAldrich, United States). 1 μg of total RNA generated first-strand cDNA. iQTM SYBR Green Supermix (Bio-Rad, United States) conducted RT-PCR in accordance with the recommendations of the manufacturer. The following primer sequences were acquired from Ruibiotech (Guangzhou, China) for this study’s targeted genes: PLCG1 (forward 5′- GGA AGA CCT CAC GGG ACT TTG -3′, reverse 5′-GCG TTT TCA GGC GAA ATT CCA-3′), PYCARD (forward 5′-TGG ATG CTC TGT ACG GGA AG-3′, reverse 5′- CCA GGC TGG TGT GAA ACT GAA-3′), CAPS8 (forward 5′- GTT GTG TGG GGT AAT GAC AAT CT -3′, reverse 5′- TCA AAG GTC GTG GTC AAA GCC-3′), NOD1 (forward 5′- TGA CAA GGT CCG CAA AAT TCT -3′, reverse 5′- ACA GCA CGA ACT TGG AGT CAC -3′), NOD2 (forward 5′- CAC CGT CTG GAA TAA GGG TAC T-3′, reverse 5′- TTC ATA CTG GCT GAC GAA ACC -3′) β-actin (Forward: 5′-CGA GCA CAG AGC CTC GCC TTT GCC-3′, Reverse: 5′-TGT CGA CGA CGA GCG CGG CGA TAT-3′). The primers of the PRGs scores-related genes are as follows: Expression data were normalized to the geometric mean of the housekeeping gene β-actin and calculated as 2^−^Δ^ΔCT^

## 3 Results

### 3.1 Identification of differentially expressed PRGs between normal and tumor tissues

**FIGURE 1:**
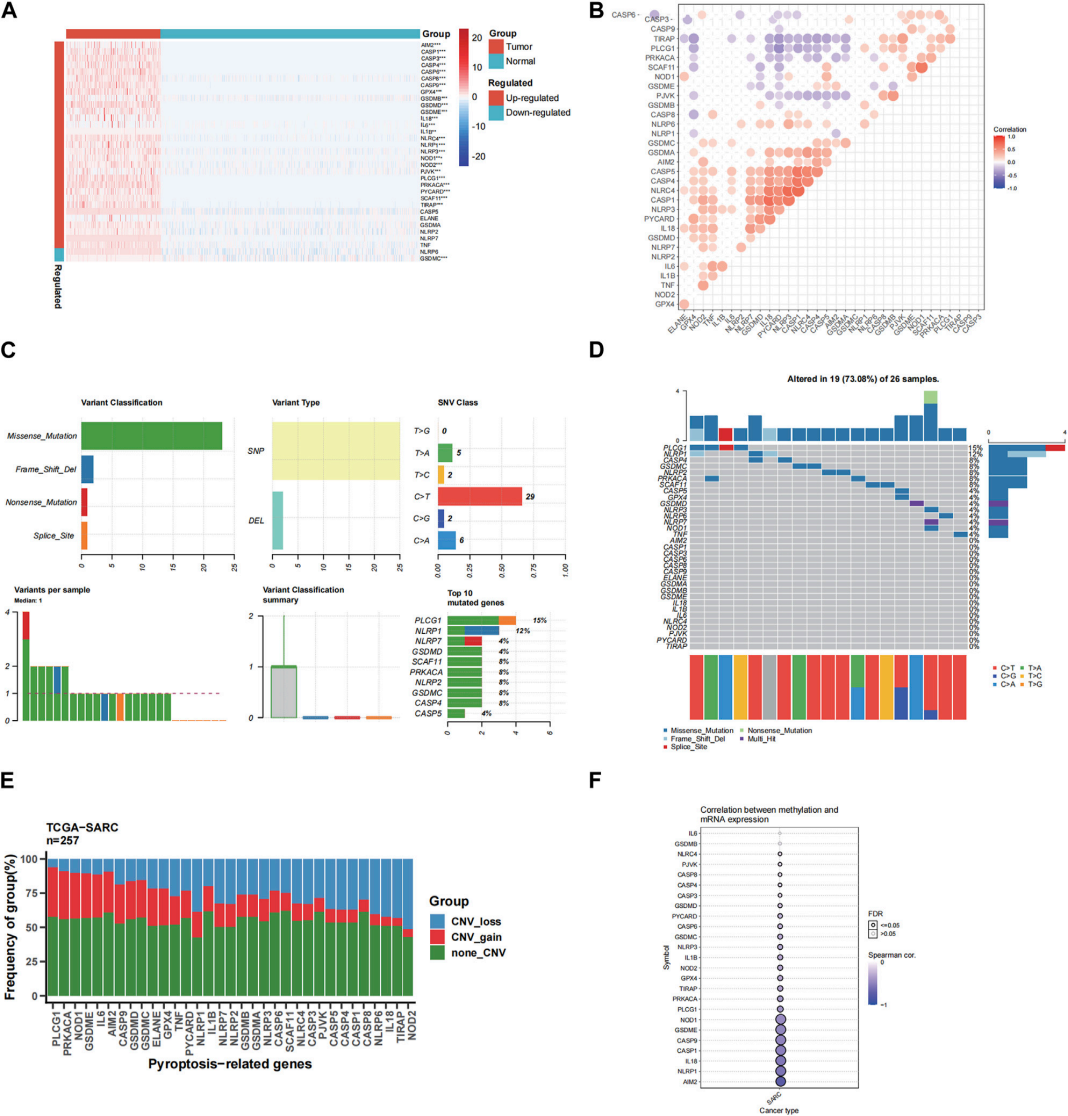
The expressions of the 33 pyroptosis-related genes and their interactions. **(A)** Heatmap (blue: low expression level; red: high expression level) of the pyroptosis-related genes between the normal (N, blue) and the tumor tissues (T, red). The *p* values were showed as: **p < 0.01; ***p < 0.001. **(B)** The correlation network of the pyroptosis-related genes (red: positive correlation; purple: negative correlation. The intensity of the color reflects the strength of the relevance). **(C)** Summary of SNV of differently expressed FRGs in STS. **(D)** SNV onplot of 26 PRGs in the TCGA-SARC cohort. **(E)** The CNV status of 26 PRGs in the TCGA-SARC cohort. **(F)** The correlation between 26 PRGs mRNA expression level with methylation rate.

Supplementary Figure S1 shows the flowchart of our study. Using the GTEx and TCGA datasets, the PRGs were compared in 864 samples of normal tissue and 263 samples from STS. There was a total of 26 PRGs found (all <0.01). Among them, 25 genes were significantly upregulated in STS, while GSDMC was enriched in normal tissues (Figure 1A). Moreover, the correlation network encompassing all PRGs was depicted in Figure 1B (red: positive correlation; purple: negative correlation).

### 3.2 Landscape of single nucleotide variation (SNV), copy number variation (CNV) and methylation

We first analyzed the association between PRGs and SNV, CNV and methylation in STS. We spotted that missense mutation, SNP, and C > T were the most frequent styles of SNV, among the differentially expressed PRGs (Figure 1C). In addition, PLCG1 and NLRP1 were the two genes with the highest rank of SNV mutations, accounting for 15% and 12% of all mutation cases, respectively (Figure 1C). The detailed SNV map showed that only 19 genes altered for STS samples (Figure 1D). As for the CNV of 26 PRGs in STS, we found that CNV amplification or deletion existed in all FRGs, especially in PLCG1, shown in Figure 1E. And the methylation rate of AIM2 and NLRP1 is negatively correlated with mRNA expression level (Figure 1F).

### 3.3 Development of prognostic gene model in the TCGA cohort

**FIGURE 2:**
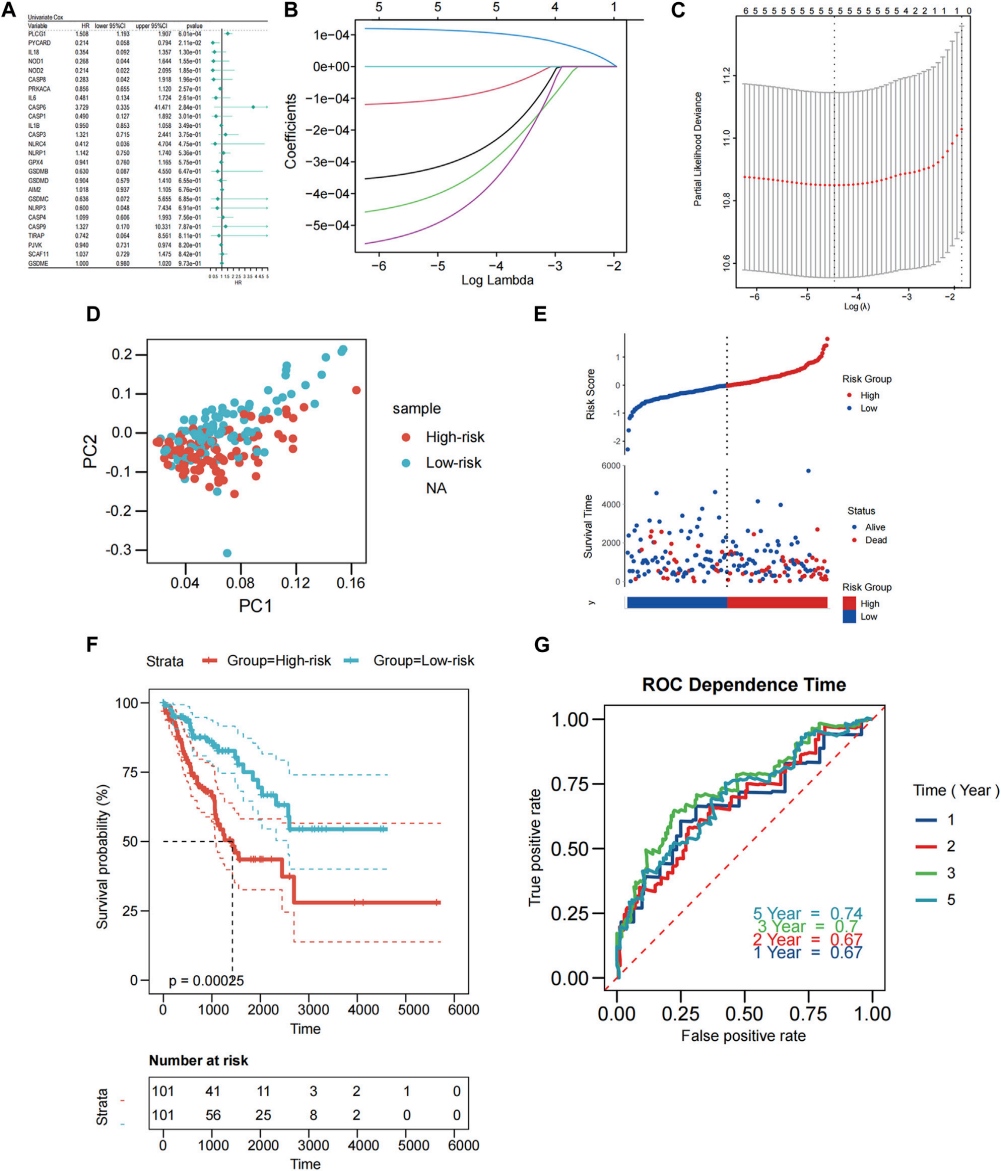
Construction of PRGs-risk model in the patients from TCGA cohort. **(A)** Univariate cox regression analysis of OS for each pyroptosis-related gene, and 5 genes with *p* < 0.2. **(B)** LASSO regression of the six OS-related genes. **(C)** Cross-validation for tuning the parameter selection in the LASSO regression. **(D)** PCA plot for STS based on the risk score. **(E)** Scatterplots in the top and bottom panels illustrate the distribution of the risk score and survival status of patients in the TCGA cohort, respectively (low-risk population: on the left side of the dotted line; high-risk population: on the right side of the dotted line). **(F)** The Kaplan-Meier curves of OS for patients in the low-risk and high-risk groups. **(G)** The time-dependent ROC curves demonstrated the predictive efficiency of the risk score.

This study associated 202 SARC samples to patients providing full survival information. The univariate Cox regression analysis was performed to conduct a preliminary screening of the genes related with survival. Six genes (PLCG1, PYCARD, IL18, NOD1, NOD2, CASP8) that met the criteria of *p* < 0.2 were maintained for further analysis. Among them, 5 genes (PYCARD, IL18, NOD1, NOD2, CASP8) were protective genes with hazard ratios (HRs) < 1, while the PLCG1 was associated with increased risk (HRs >1) (Figure 2A). By performing the LASSO regression analysis, a 5-gene signature was constructed according to the optimum λ value (Figure 2B,C). The PRGs-risk score was calculated as follows: risk score = (11.06E-5* PLCG1.EXP) + (28.01E-5 * CASP8.EXP) + (−35.09E-5 * NOD1.EXP) + (−43.24E-5 *NOD2.EXP) + (−9.01E-5 * PYCARD.EXP).

Based on the risk scoring formula’s median scores, the 202 samples were equally split into two: low PRGs-risk and high PRGs-risk. PCA findings indicated that patients with varying risks were effectively separated into two (Figure 2D). Patients in the low-PRGs-risk group lived for longer periods of time and had a higher proportion of survivors (Figure 2E). Between the high- and low-PRGs risk groups, a substantial discrepancy exists (*p* < 0.001, Figure 2F), but not in RFS (*p* = 0.052, Supplementary Figure S2A–B). ROC analysis assessed the sensitivity and specificity of the prognostic model. For OS, we discovered that the area under the receiver operating characteristic curve (AUC) was 0.67, 0.67, 0.7, and 0.74 for 1-year, 2-year, 3-year, and 5-year models, respectively (Figure 2G).

### 3.4 External validation of the risk models

**FIGURE 3:**
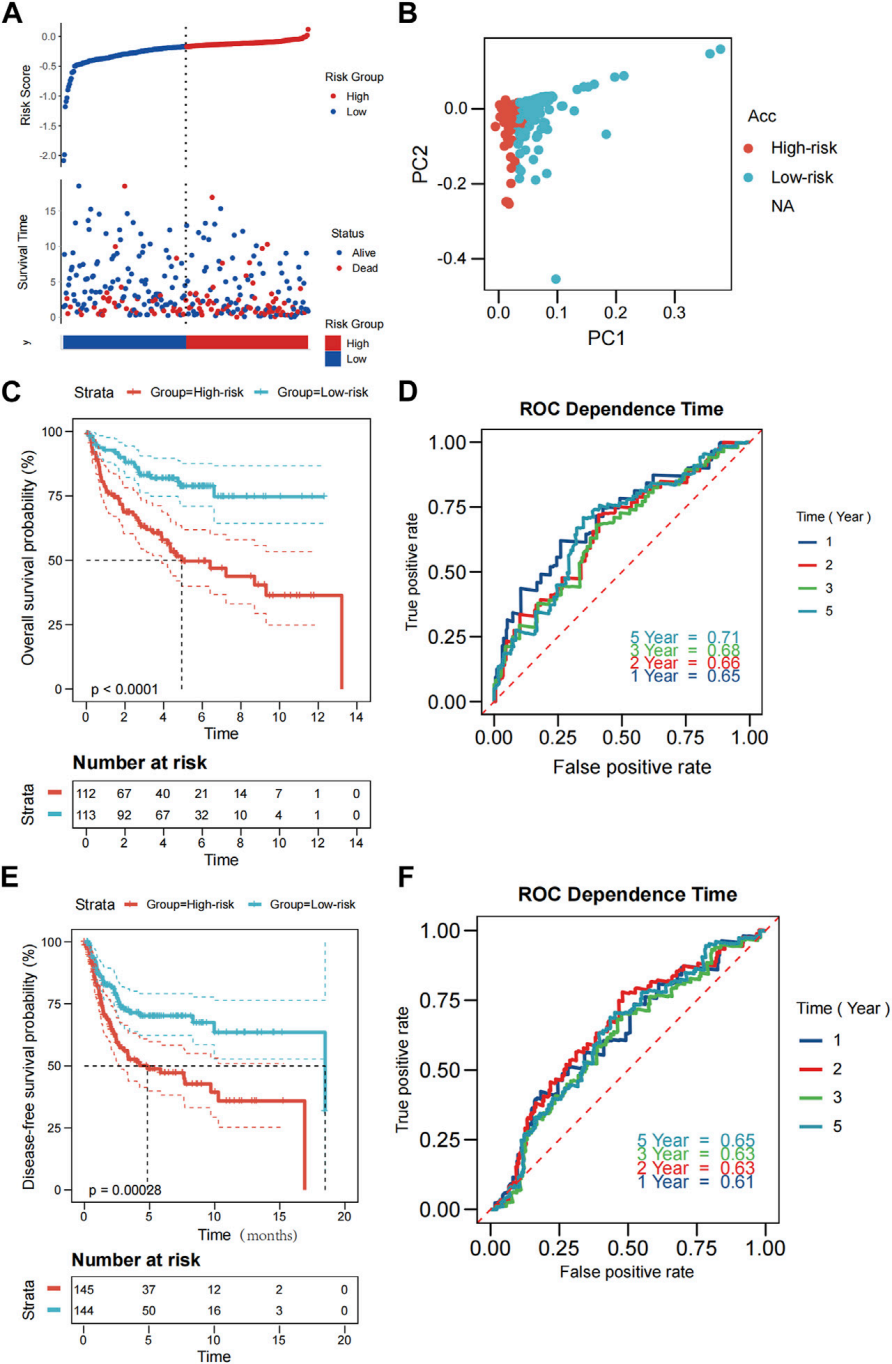
Validation of the PRGs-risk model in the GEO cohort. **(A)** Distribution of patients in the GEO cohort based on the median risk score in the TCGA cohort and the survival status for each patient (low-risk population: on the left side of the dotted line; high-risk population: on the right side of the dotted line. **(B)** PCA plot for STS. **(C, E)** The Kaplan-Meier curves for comparison of the OS (GSE30929 and GSE63157) and DFS(GSE21050) between the patients in the low-risk and the high-risk groups. **(D,F)** The time-dependent ROC curves of OS and DFS in the GEO cohort.

External validation was performed on 290 patients from GSE21050 for RFS and 226 patients from GSE30929 and GSE63157 for OS. Prior to further analysis, we normalized gene expression data using the “Combat” program to eliminate batch effects. The TCGA cohort algorithm was applied to produce the risk scores, categorizing the patients into low- and high-risk categories from their median scores. Those belonging to the former group had a longer lifespan and smaller rate of mortality compared to the latter group (Figure 3A). The PCA results indicated that the two groupings were sufficiently distinct (Figure 3B). Additionally, Kaplan-Meier analysis revealed significant differences in OS and DFS between the low- and high-risk groups ((both *p* < 0.001; Figure 3C; Figure 3E). As illustrated in Figure 3D and Figure 3F, our model exhibited a high predictive value (AUC for OS: 0.65 for 1 year, 0.66 for 2 years, 0.68 for 3 years, 0.71 for 5 years; AUC for RFS: 0.61 for 1 year, 0.63 for 2 years, 0.63 for 3 years, 0.65 for 5 years).

### 3.5 Independent prognostic value of the PRGs-risk scores

**FIGURE 4:**
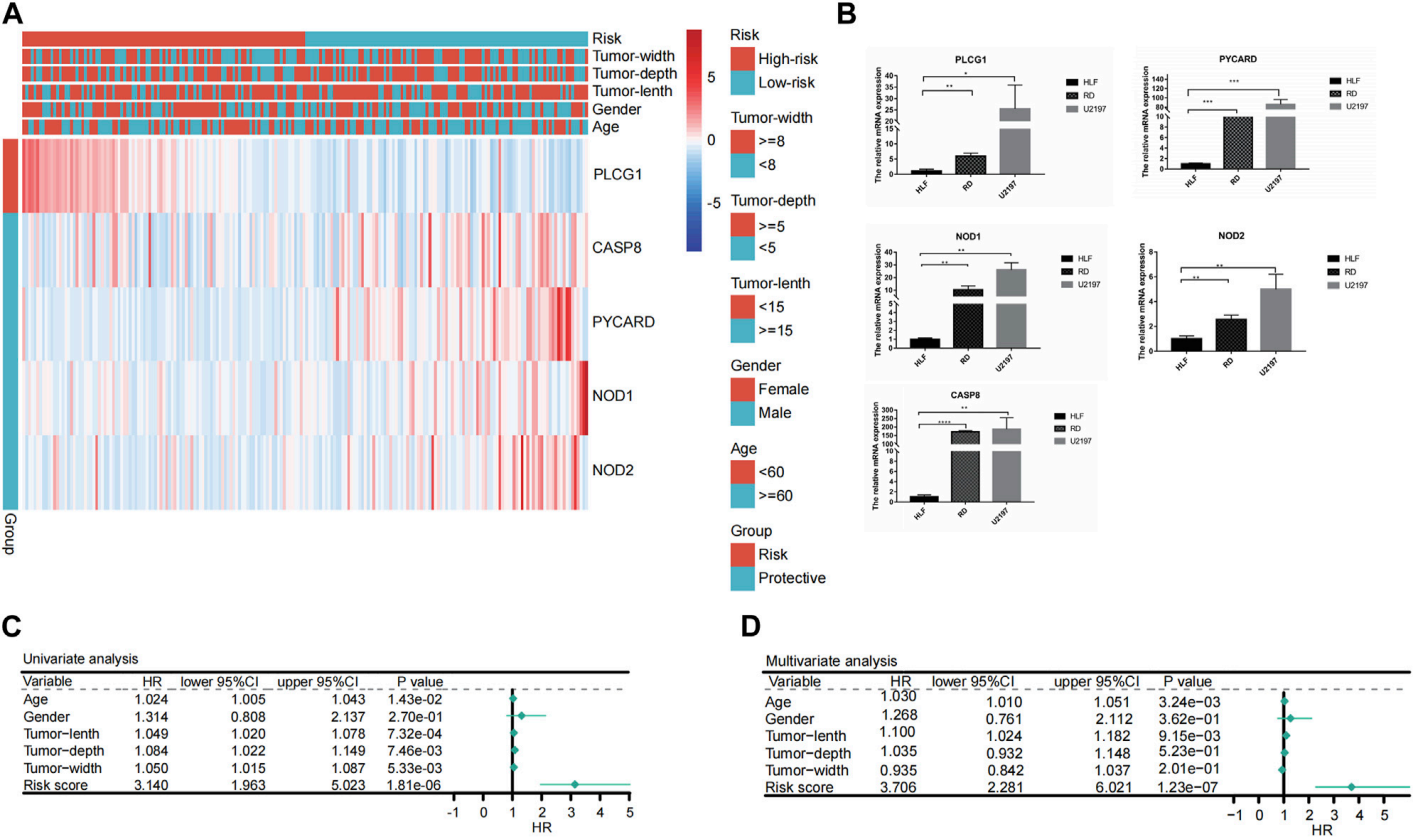
Univariate and multivariate Cox regression analyses for the PRGs-risk score. **(A)** Heatmap depicting five genes different expression in high- and low- PRGs-risk and the corrlection with clinical characteristics. **(B)** Results of qRT-PCR analysis. **(C)** Univariate analysis of the clinicopathological features and the risk score for the TCGA cohort. **(D)** Multivariate analysis of the clinicopathological features and the risk score for the TCGA cohort.

**FIGURE 5:**
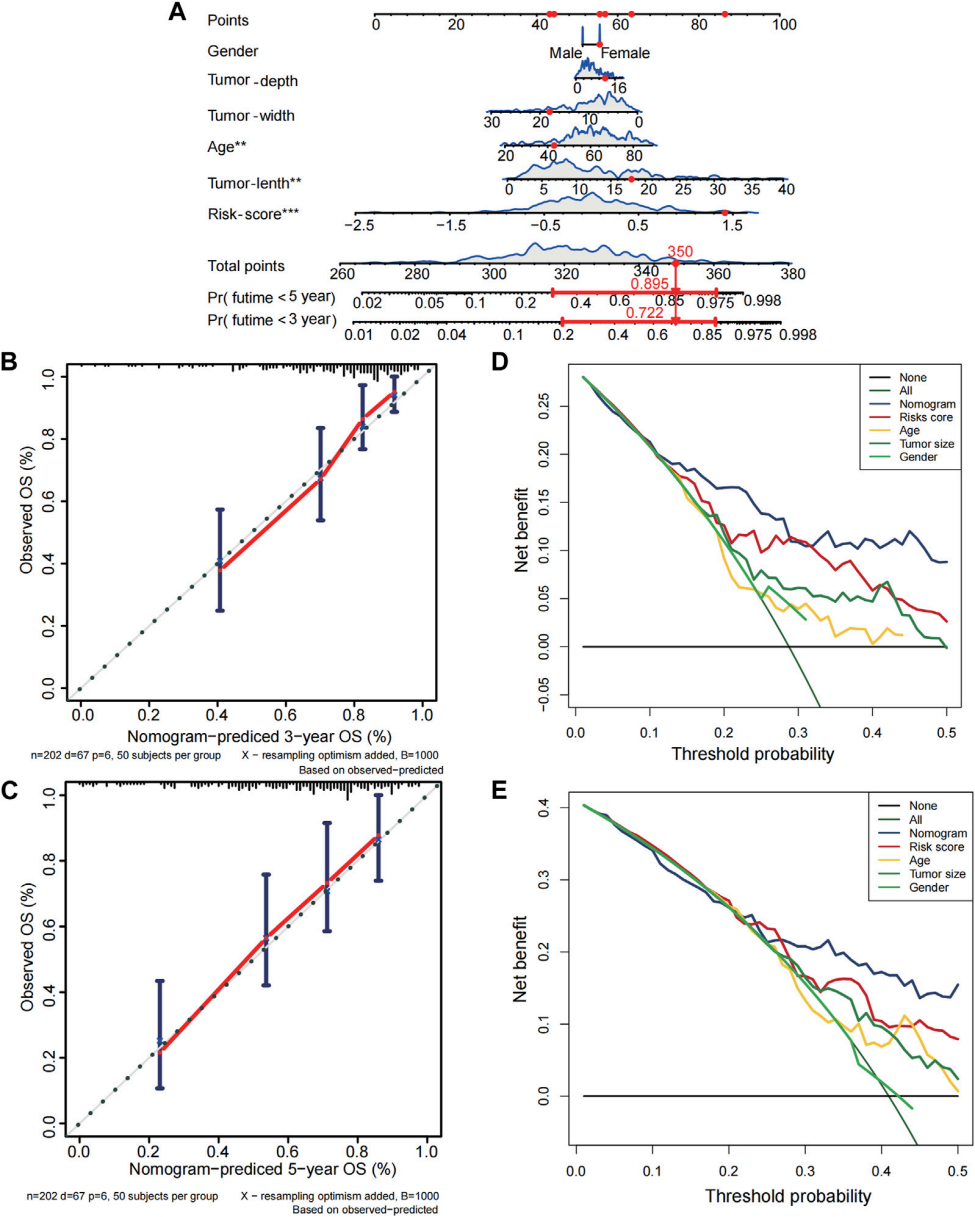
Establishment of nomogram for soft tissue sarcoma patients in the TCGA cohort. (**A)** The nomogram for predicting overall survival(OS) of soft tissue sarcoma patients based on combination of risk score and clinical features. **(B–C)** The calibration curves of nomogram regarding 3 years-OS and 5 years-OS. **(D–E)** The decision curve analyses for nomogram. X-axis represents threshold probabilities, and Y-axis measures net benefit.

Only the TCGA-SARC cohort was utilized to test the association between clinical features and PRGs-risks due to insufficient clinical data from the GEO database. Upon examination of the correlation between PRGs-risk groups and various STS clinical parameters, there was insignificant relationship between PRG-risk scores abd the patients’ gender, age, and tumor depth and size (Figure 4A). Furthermore, the genes’ mRNA expression levels with respect to the risk scores were considerably elevated in sarcoma cell lines compared to HLF, based on the qRT-PCR findings (Figure 4B). Afterward, it was determined whether PRGs-risk scores may be independent prognostic factors through univariate and multivariate cox regression analyses. The high PRGs-risk score was strongly related with poor survival in the TCGA-SARC cohort, according to the univariate cox regression analysis (HR = 3.140, 95%CI:1.963–5.023; Figure 4C). It was further suggested by the multivariate study that the PRGs-risk scores could be independent predictive factors after regulating the other confounding elements (HR = 3.706, 95%CI:2.281–6.021) for patients with STS (Figure 4D).Additionally, a nomogram was created to demonstrate quantitative prediction of OS for STS patients (Figure 5A). The calibration curves for three- and 5-year survival tended to adhere to the 45°standard line, showing that the nomogram model performed well in terms of prediction (Figures 5B,C). The DCA analysis revealed that the nomogram model provides the greatest net benefit for decision making across the majority of thresholds. (Figures 5D,E).

### 3.6 Comparison of the immune state between different PRGs-risk subgroups

**FIGURE 6:**
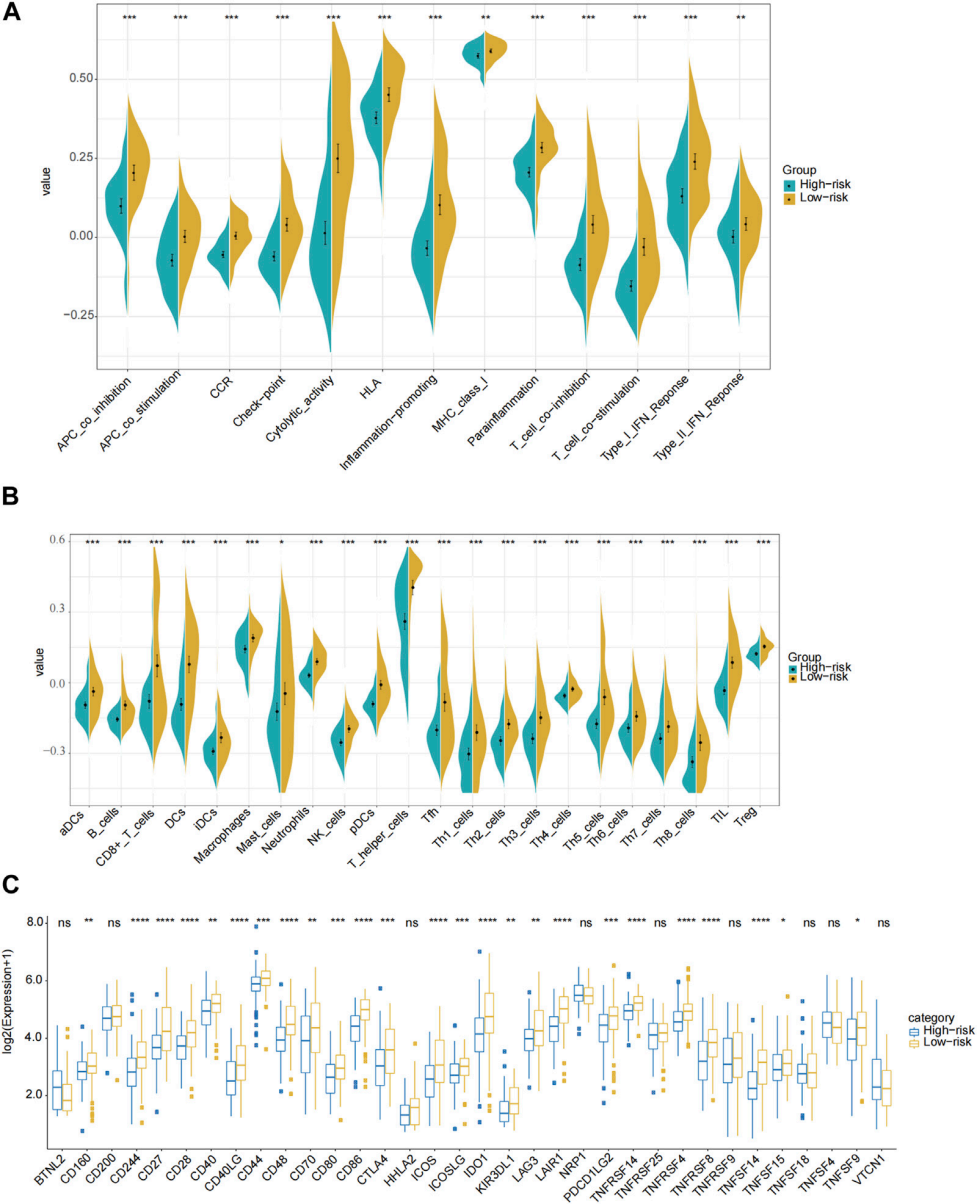
Comparison of the ssGSEA scores for immune pathways, immune cells and immune check points in the TCGA cohort. **(A)** Comparison of the enrichment scores of 13 immune-related pathways between the low-risk (yellow box) and the high-risk (blue box) groups. **(B)** Comparison of the infiltration of 24 types of immune cells between the low-risk (yellow box) and the high-risk (blue box) groups. **(C)** Comparison of the expression of different immune checkpoints between the low-risk and the high-risk groups in the TCGA cohort. P values were showed as: ns not significant; * *p* < 0.05; ** *p* < 0.01; *** *p* < 0.001; **** *p* < 0.0001.

Based on the PRGs, analyses of GO enrichment and KEGG pathways were subsequently conducted. Accordingly, PRGs were typically associated with cheekiness-mediated signaling pathways, immune response, and chemotaxis of inflammatory cells (Supplementary Figure S3A–B). We then evaluated the 24 kinds of immune cells’ enrichment scores, typical immunological checkpoints, and activity of 13 immune-related pathways for both risk groups and cohorts based on functional analysis. In this case, R package “gsva” was utilized to undertake ssGSEA or single-sample gene set enrichment analysis. Additionally, we assessed the activity of immune-related pathways. In the TCGA cohort, the high-risk subgroup with lower levels of immune cell infiltration (especially CD8^+^ T cells, neutrophils, natural killer cells and tumor-infiltrating lymphocytes) and immunosuppressed state than the low-risk subgroup (Figures 6A–C). Similar conclusions were drawn when analyzing the immune state of the GEO cohort (Supplementary Figure S4A–C).

### 3.7 Patients with low PRGs-risk scores were more likely to benefit from anti-PD-L1 therapy

**FIGURE 7:**
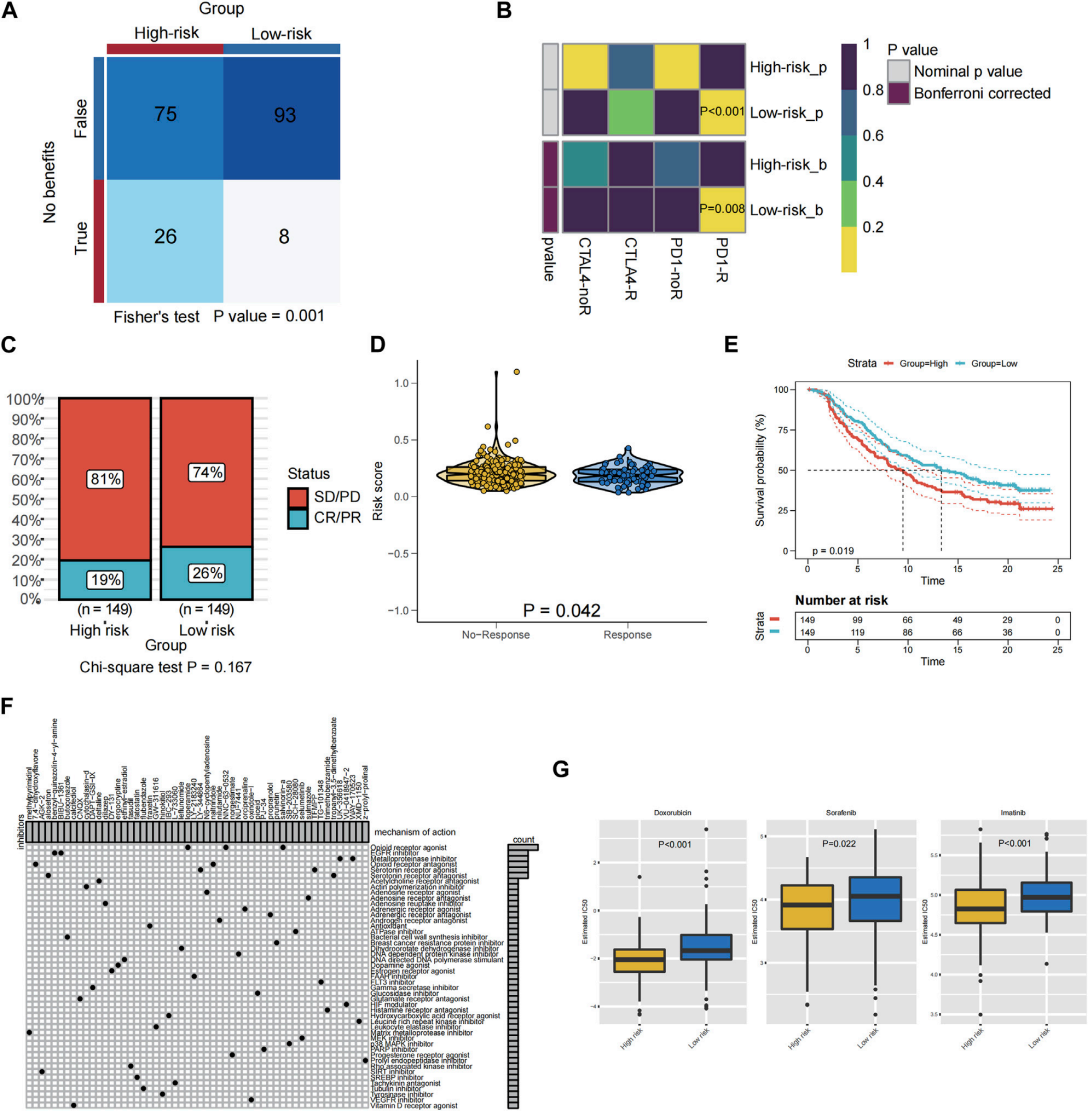
Relationship between PRGS risk score and antitumor drug sensitivity and immunotherapy response. **(A)** Compared different PRGs-risk group benefit from ICIs according to TIDE. **(B)** Subclass mapping analysis for predicting the likelihood of response to ICI therapy of patients with different PRGs-risk score. **(C)** The proportion of patients in the IMvigor210 cohort with different responses (PR + CR vs. PD + SD) to PD-L1 blockade immunotherapy. **(D)** The difference in the PRGs-risk score between the non-response and the response groups in the IMvigor210 cohort. **(E)** Kaplan-Meier graphs depicting patients’ overall survival (OS) in the high-risk (red) and low-risk (blue) categories following PD-L1 immunotherapy in the IMvigor210 cohort. **(F)** Potential targets of compounds and corresponding signaling pathway were employed in the CMAP database. **(G)** The boxplot showed a significant difference in doxorubicin (*p* < 0.001), sorafenib (*p* = 0.022), and imatinib (*p* < 0.001) IC50 values predicted by the pRRophetic method using the GDSC database between the high PRGs-risk group and the low PRGs-risk group.

Patients with advanced STS may benefit from ICIs, according to previous clinical trails, and some markers may be able to predict whether or not they would respond to immunotherapy. In the TIDE algorithm, low PRGs-risk patients would more likely benefit from immunotherapy than high PRGs-risk ones (*p* < 0.05, Figure 7A). In addition, patients with low PRGs-risk demonstrated a more promising anti-tumor impact when treated with anti-PD-1 treatments (Figure 7B, Bonferroni correction *p* < 0.05). Transcriptomic data from uroepithelial carcinoma patients treated with the anti-PD-L1 antibody atezolizumab (IMvigor210) were then evaluated to verify the response of the two PRGs-risk groups. In the low-risk group, the proportion of patients with partial response (PR) and complete response (CR) was greater than in the high PRGs-risk group. (low-risk group vs. high-risk group: 26% vs. 19%, *p* = 0.167; Figure 7C). Notably, the non-respond group had a higher PRGs-risk score than the response group (*p* = 0.042, Figure 7D). Furthermore, high PRGs-risk patients lived much shorter than low PRGs-risk ones (*p* = 0.019, Figure 7E).

### 3.8 The potential small molecule inhibitors for STS patients with different PRGs-risk group

For advanced STS, chemotherapy and targeted therapy are the mainstays of treatment, however some patients did not benefit from them. Therefore, it is vital to identify those patients who were more responsive to chemotherapy and targeted medications, which may assist clinicians to employ the optimal strategy.CMAP analysis was used to find small molecular drugs for STS patients based on PRGs in order to explore prospective molecular therapeutics. A total of 54 kinds of inhibitors with 47 types of mechanisms of action (MOA) were identified (Figure 7F). Examples include the opioid receptor agonist salvinorina, the EGFR inhibitor BIBU 1361, and the metalloproteinase inhibitor UK356618, which were predicted to be potentially useful for the treatment of STS.

And the pRRophetic algorithm was utilized in conjunction with the GDSC database to assess the response of two distinct PRGs-risk groups to chemotherapeutic and targeted medicines licensed for STS patients. Remarkably, the estimated IC50 levels of doxorubicin (*p* < 0.001), sorafenib (*p* = 0.022), and imatinib (*p* < 0.001) were distinctly lower in the patients of high-risk compared to those of low PRGs-risk group, suggesting that patients in the high-risk subtype were more sensitive to chemotherapy and targeted therapy(sorafenib and imatinib) (Figure 7G).

## 4 Discussion

As a newly discovered type of programmed cell death, pyroptosis is characterized by cell swelling, morphological enlargement, and inflammasome release (Bergsbaken et al., 2009). Various investigations have indicated the dual function of pyroptosis in cancer development. Pyroptosis cell-secreted inflammatory substances may facilitate the malignant transformation of normal cells (Wang et al., 2016a; Wang et al., 2016b), while on the other hand, pyroptosis itself or triggered by other factors can inhibit tumor progression or metastasis (Le et al., 2020; Tan et al., 2020). To date, it remains unknown whether PRGs are associated with the prognosis and development mechanisms of STS. This research systematically examined the expressions of 33 PRGs in STS and their connection with prognosis.

In this study, we conducted a comprehensive analysis of the expressions and prognostic values of PRGs in STS. Our results indicated that, except for NLRP6 and GSDMC, the expressions of most PRGs were higher in tumor tissues compared to normal tissues. Among these, phospholipase C gamma 1 (PLCG1), a membrane-associated enzyme involved in cell growth and differentiation mediated by leucine kinase receptor signaling pathway, has been shown to induce pyroptosis by increasing the activity of GSDMD via intracellular calcium signaling, which is implicated in fatal infection (Kang et al., 2018; Liu et al., 2020). However, little is known about the association between PLCG1-mediated pyroptosis and malignancies. Our findings showed that PLCG1 was highly expressed in STS and was associated with low survival, possibly due to negative regulation of pyroptosis.

Currently, it is widely recognized that NOD1 and NOD2, which are important members of the intracellular PRR family, participate in regulating innate immunity *in vivo* and inducing pyroptosis together with NLRP3 (Jamilloux et al., 2013; Shi et al., 2020). Caspase-8 was previously thought to be the main enzyme inducing apoptosis, but recent studies have demonstrated its potential in facilitating gasdermin C and gasdermin D cleavage, thereby inducing pyroptosis (Sarhan et al., 2018; Fritsch et al., 2019; Hou et al., 2020). Moreover, studies have revealed that α-KG can induce pyroptosis to inhibit tumor growth by activating caspase-8 and cleaving gasdermin C, suggesting that caspase-8-mediated pyroptosis may enhance the efficacy of antitumor drugs (Zhang et al., 2021). Furthermore, we performed multivariate analysis and external validation of the scoring system, and the data indicate that the risk signature may serve as an independent predictor for STS. In addition, we established and validated a novel nomogram based on PRGs in STS patients for the first time, and demonstrated its high accuracy in predicting the survival of STS patients.

To date, numerous studies have demonstrated that chemotherapy or targeted drugs, such as cisplatin, lobaplatin, and sorafenib, can augment their anti-tumor activity by inducing pyroptosis of tumor cells, which leads to improved prognosis for patients (Hage et al., 2019; Zhang et al., 2019; Chen et al., 2020). However, most chemotherapy drugs achieve their anti-tumor effects by promoting tumor cell apoptosis rather than pyroptosis. Consequently, there is a growing interest in discovering pyroptosis-inducing drugs. Small molecule inhibitors, including DD8/9 inhibitors and apurinic/apyrimidinic endonuclease 1 (APE1) inhibitors, have been shown to induce pyroptosis and enhance the prognosis of patients with malignancies (Johnson et al., 2018; Long et al., 2021). In this study, we further evaluated the effectiveness of drugs approved for STS patients, including chemotherapy drugs and targeted drugs, by predicting their efficacy in cases with varying scores of pyroptosis characteristics. Our results demonstrated that only adriamycin, sorafenib and imatinib could benefit patients in the high-risk group. Moreover, we screened several specific small molecule inhibitors using the CAMP database, which suggests that some of them may enhance anti-tumor activity by inducing pyroptosis. Further *in vivo* and *in vitro* studies are needed to identify more effective targeted STS-based medications.

The tumor microenvironment plays a crucial role in determining the progression of STS and the appropriate therapeutic response. Petitprez et al. divided 608 patients with STS into 5 groups based on the different components of their immune microenvironment and found that patients with high immune cell infiltration had higher expression levels of various immune checkpoints, including PD-L1, PD-L2, CTLA-4, and TIM-3, and had a better clinical prognosis (Petitprez et al., 2020). In this study, we examined the functional signaling pathways and found that the varying risks of STS patients with different PRGs were associated with specific immune-related pathways, suggesting that these PRGs could be involved in STS immune regulation. We also found that the high-risk group had lower levels of impaired immune-related pathway activities and immune cell infiltration compared to the low-risk group. These findings indicate that the microenvironment of STS patients in the high PRGs-risk group exhibited an immunosuppressive condition, potentially a primary cause for the poor prognosis among such patients. Further studies are necessary to explore the potential of PRGs as therapeutic targets for STS patients with an immunosuppressive microenvironment.

Petitprez et al. have reported that STS patients with significant immune infiltration exhibit increased expression of immunological checkpoints, such as PD-L1, PD-L2, CTLA-4, and TIM-3, and better clinical outcomes (Petitprez et al., 2020). Our study found that high-risk patients with low PRGs expression had a higher level of immunological checkpoint expression, which is consistent with previous research. High PD-L1 expression is considered to be a marker of better response to immunotherapy. This study utilized TIDE and IMvigor210 cohorts to predict the benefit of immunotherapy in both groups and found that low-risk patients potentially benefit more from anti-PD-1 treatment, presumably due to their increased PD-L1 expression. It further revealed that the anti-PD-1 antibody is more suitable for STS patients with attributes similar to the low-risk group. Thus, the PRGs-based model may serve as a reliable marker to predict the effectiveness of immunotherapy.

This research categorized STS patients based on PRGs and further demonstrated that the model provides a valid scheme to predict prognosis and identify therapeutic groups. However, the study is limited by its retrospective nature and the limited number of STS patients available in the databases, which may lead to selection bias. Therefore, large-scale and multicenter studies are necessary to verify the reliability of the current model and optimize its applicability. Additionally, *in vivo* and *in vitro* research is needed to validate PRGs and examine relevant processes.

## 5 Conclusion

In conclusion, this study provides a reliable diagnostic tool for STS prognosis. The PRGs model proposed in this research is an independent marker that can effectively evaluate the prognosis of STS patients, facilitating the establishment of individualized and targeted therapeutic approaches.

## Figures and Tables

**Table:** 

**STS**	Soft tissue sarcoma
**TCGA**	The cancer genome atlas
**LASSO**	Least absolute shrinkage and selection operator
**ROC**	Receiver operating characteristic curve
**OS**	Overall survival
**GEO**	The gene expression omnibus database
**TIDE**	The tumor immune dysfunction and exclusion
**CMAP**	The connectivity map
**NLRP3**	Nucleotide binding domain-like receptor 3
**PRGs**	Pyroptosis-related genes
**DFS**	Disease-free survival
**PCA**	Principal component analysis
**DCA**	Decision curve analysis
**GO**	Gene ontology
**KEGG**	Kyoto encyclopedia of genes and genomes
**ssGSEA**	Single-sample gene set enrichment analysis
**GDSC**	The genomics of drug sensitivity in cancer
**PPI**	The protein - protein interaction
**AUC**	The area under the receiver operating characteristic curve
**ICIs**	The immune checkpoint inhibitors
**HRs**	Hazard ratios
**PR**	Partial response
**CR**	Complete response
**MOA**	The mechanisms of action
**APE1**	Apurinic/apyrimidinic endonuclease 1
**PD-L1**	Programmed cell death-1
**PD-L2**	Programmed cell death-1
**CTLA-4**	Cytotoxic T lymphocyte-associated antigen-4
**TIM-3**	T cell immunoglobulin and mucin domain-containing protein 3
**PYCARD**	PYD and CARD domain containing
**CASP1**	Cysteine-aspartic acid protease-1
**IL1B**	Interleukin 1 beta
**PLCG1**	Phospholipase C gamma 1
**IL18**	Interleukin 18
**NOD1**	Nucleotide binding oligomerization domain containing 1
**NOD2**	Nucleotide binding oligomerization domain containing 2
**CASP8**	Cysteine-aspartic acid protease-8
**NLRP6**	NLR family pyrin domain containing 6
**GSDMC**	Gasdermin C
**NCCN**	The national comprehensive cancer network

## Data Availability

The original contributions presented in the study are included in the article/Supplementary Material, further inquiries can be directed to the corresponding author.

## Supplementary material

Supplementary Figure S1 Flowchart of this study.

Supplemental Figure S2 The comparison of the RFS between the different risk groups in TCGA. **(A)** Kaplan-Meier curves for comparison of the RFS between the low-risk and the high-risk groups in the TCGA cohort. **(B)** Time-dependent ROC curves for STS in the TCGA cohort.

Supplemental Figure S3 The results of functional analysis based on the PRGs between the two groups in the TCGA cohort. **(A)** The barplot graph of KEGG pathway enrichment analysis (the longer bar meant the more genes enriched, and the increasing depth of red meant that the differences were more significant). **(B)** The bubble graph for GO enrichment analysis (the bigger bubble meant the more genes enriched, and the increasing depth of red meant that the differences were more significant; *p*-value: the adjusted *p*-value.

Supplemental Figure S4 Comparison of the ssGSEA scores of immune-related pathway activity, immune cell infiltration and immune check point expression in the GEO cohort. **(A)** Comparison of the enrichment scores of 13 immune-related pathways between the low-risk (yellow box) and the high-risk (blue box) groups. **(B)** Comparison of the infiltration of 24 types of immune cells between the low-risk (yellow box) and the high-risk (blue box) groups. **(C)** The different immune checkpoints expression levels in the low-risk (yellow box) and high-risk (blue box) groups. p values were showed as: ns not significant; * *p* < 0.05; ** *p* < 0.01; *** *p* < 0.001; *****p* < 0.0001.
