# Supplementary figures and images for: Pyroptosis-related genes prognostic model for predicting targeted therapy and immunotherapy response in soft tissue sarcoma
Source: Front Pharmacol. 2023 May 5;14:1188473. doi: 10.3389/fphar.2023.1188473 (PMC10196039; doi:10.3389/fphar.2023.1188473)

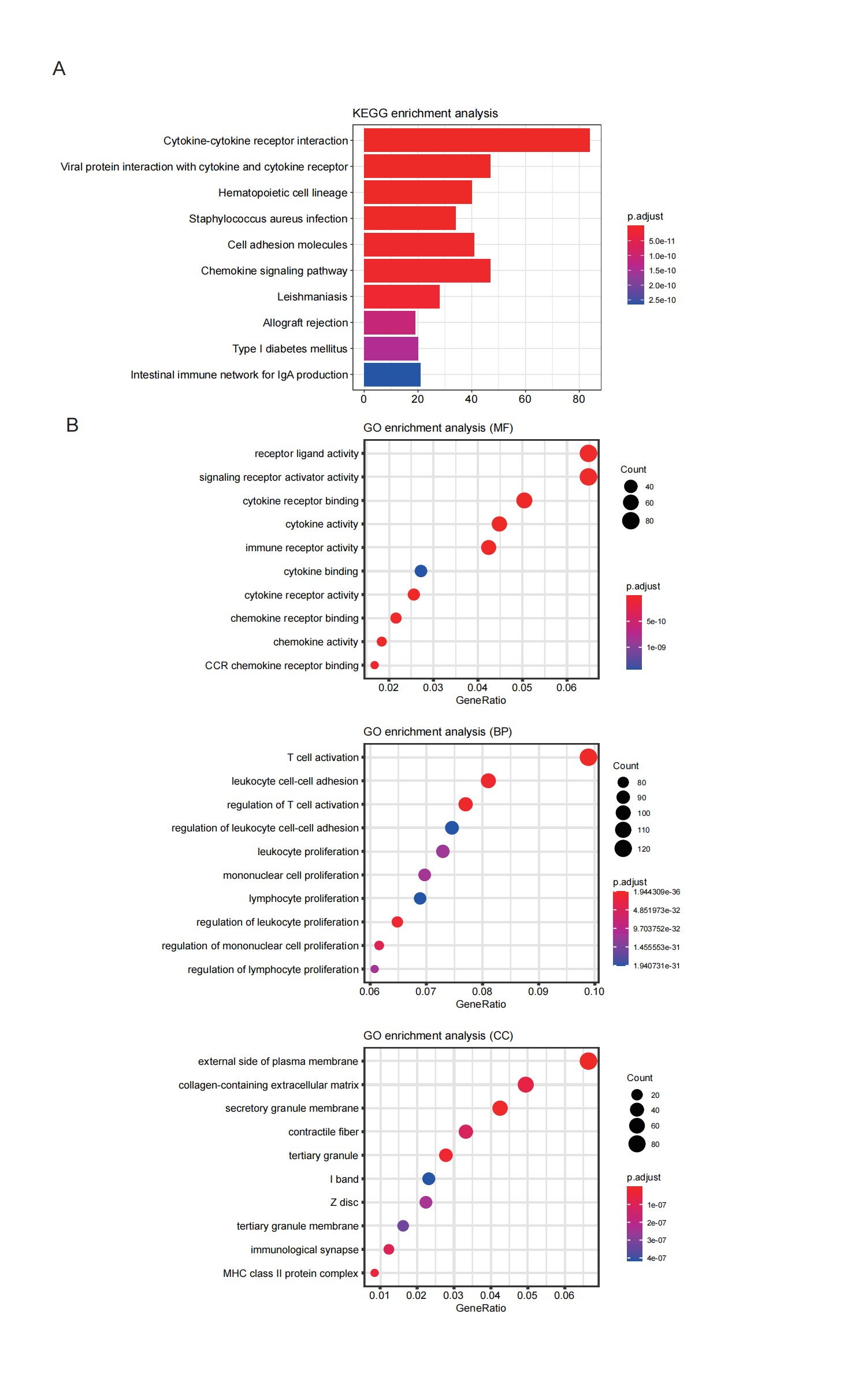

Supplement: Supplementary file 2 [file Image3.TIF]

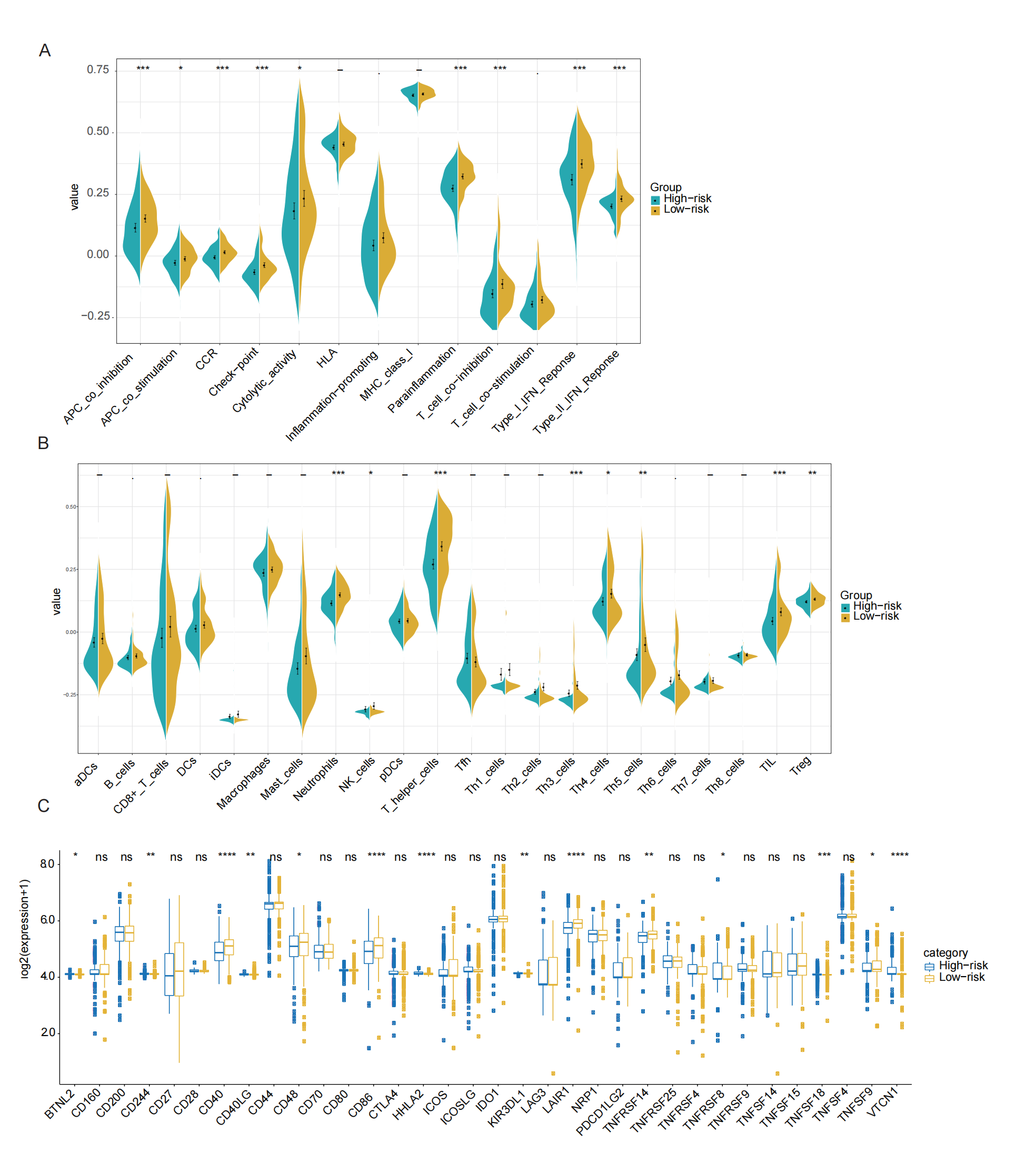

Supplement: Supplementary file 3 [file Image4.TIF]

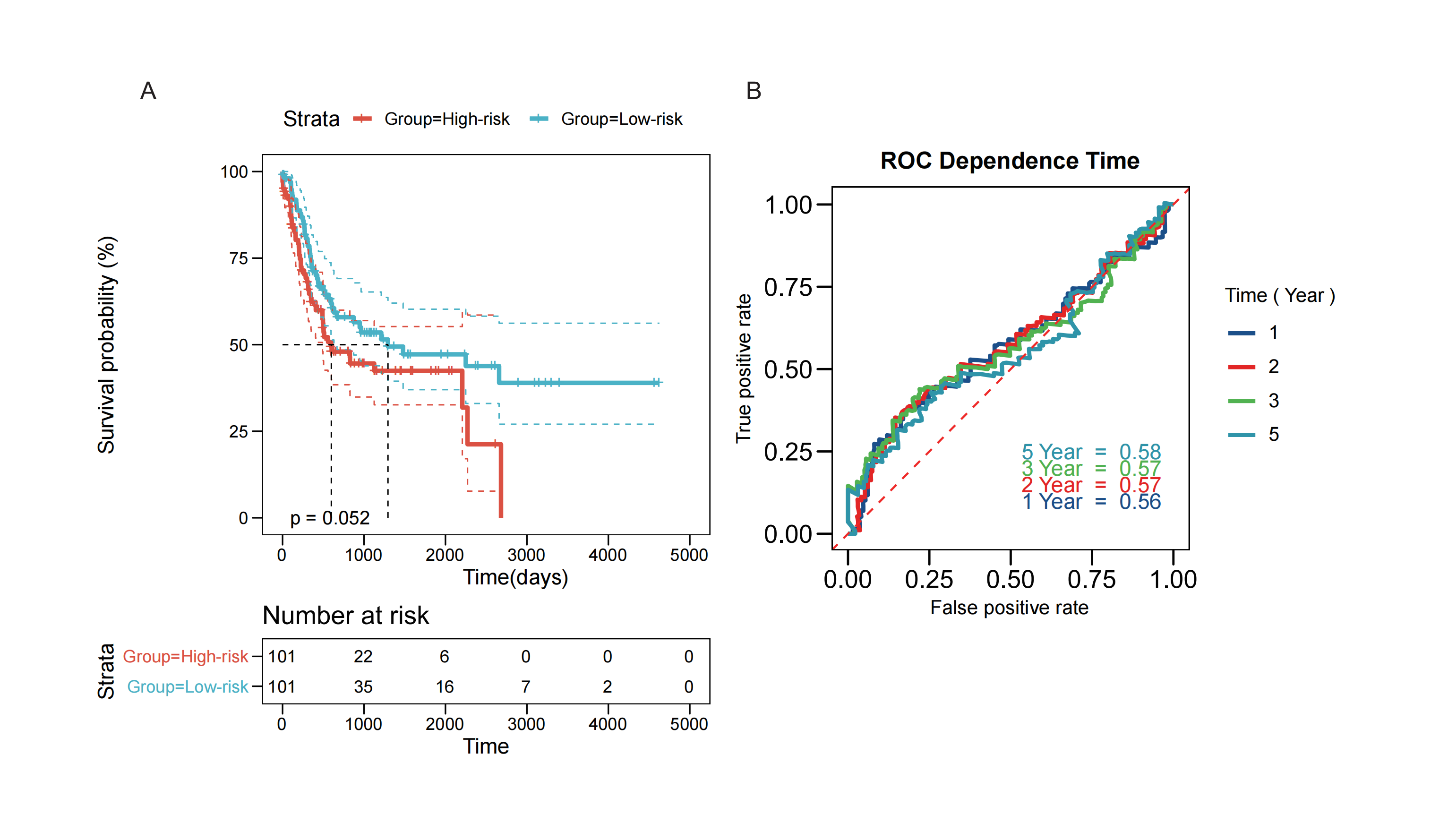

Supplement: Supplementary file 5 [file Image2.TIF]

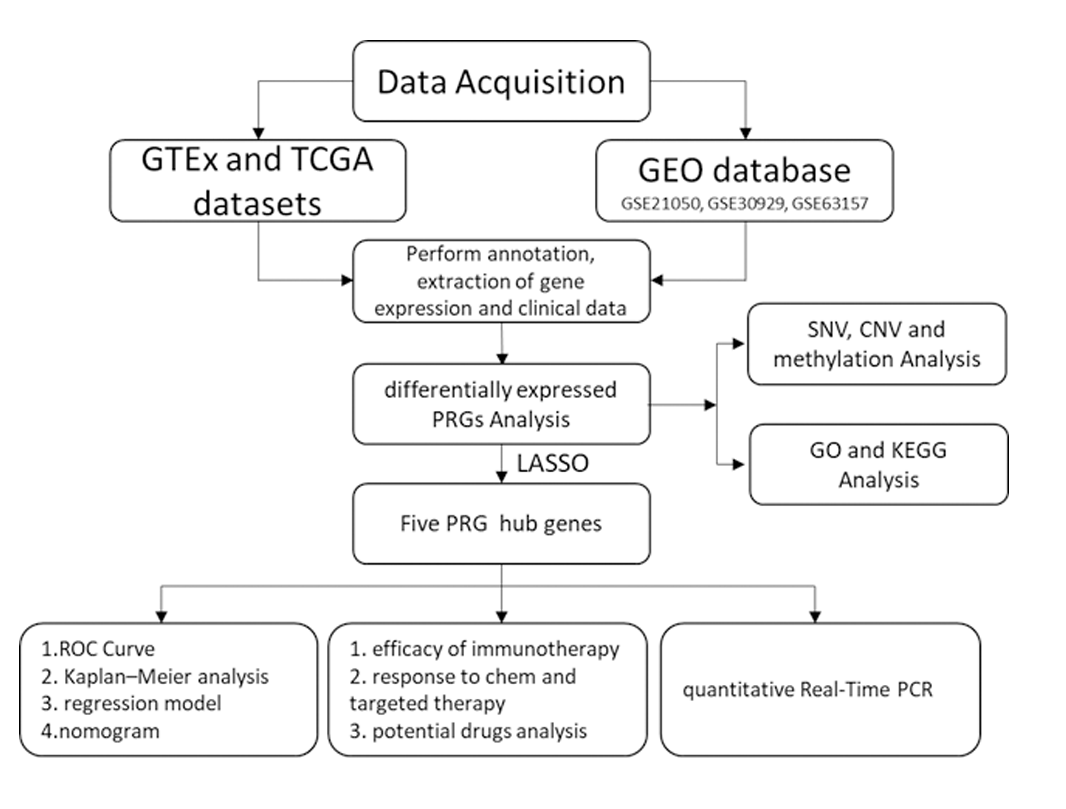

Supplement: Supplementary file 6 [file Image1.TIF]
